# 6-4 photolyase differentially modulates transcription in the vertebrate circadian clock

**DOI:** 10.1371/journal.pgen.1011971

**Published:** 2025-12-12

**Authors:** Hongxiang Li, Carina Scheitle, Xiaodi Hu, Jannis J. Kaiber, Takeshi Todo, Daniela Vallone, Reinhard Fischer, Nicholas S. Foulkes

**Affiliations:** 1 Institute of Biological and Chemical Systems, Karlsruhe Institute of Technology, Eggenstein‐Leopoldshafen, Germany; 2 Department of Microbiology, Institute for Applied Biosciences, Karlsruhe Institute of Technology, Karlsruhe, Germany; 3 Radioisotope Research Center, Institute for Radiation Science, Osaka University, Osaka, Japan; Harvard Medical School, UNITED STATES OF AMERICA

## Abstract

The cryptochrome-photolyase family, a highly conserved set of flavoproteins, mediates many direct and indirect responses to sunlight. While the photolyases are light-dependent enzymes which catalyze photoreactivation repair of UV-induced DNA damage, the cryptochromes serve as circadian clock components and photoreceptors. Do DNA repair and circadian clock functions overlap in these flavoproteins? While 6–4 photolyase (6–4phr) is well-documented to repair UV-induced 6–4 photoproducts, we demonstrate that loss of 6–4phr function in fish cells and fin clips significantly attenuates circadian rhythms of *period* gene expression. Importantly, 6–4phr represses, as well as activates transcription directed by E-box and D-box enhancer elements respectively. Furthermore, we document physical interaction between 6–4phr and Clock1/Bmal1 at multiple domains which interferes with Clock1-Bmal1 heterodimerization. In addition, 6–4phr interacts with the D-box binding transcription factor, Tef. Thus, we reveal significant overlap between DNA repair and circadian clock functions in 6–4phr.

## Author summary

This work sheds new light on the function of photolyases in vertebrates. Photolyases are highly conserved flavoproteins which have been studied extensively concerning their role in DNA damage repair. However, a potential role for photolyases in the circadian clock remains poorly understood. Here, by using fish models, we reveal that the loss of 6–4 photolyase (6–4phr) results in attenuation of circadian rhythms of clock gene expression. Furthermore, 6–4phr physically interacts with and thereby suppresses Clock1-Bmal1 mediated transactivation via the E-box enhancer. In addition, 6–4phr also increases transcriptional activation mediated by the D-box enhancer via physical interaction with Tefb. These findings point to a dual functionality for 6–4phr and provide new insight into the general functionality of photolyases in vertebrates.

## Introduction

Light represents a fundamentally important environmental factor for most living systems. Regular changes in the intensity and spectrum of sunlight underlie the day-night and seasonal cycles which dominate the environment. As a key strategy for surviving the day-night cycle, organisms have evolved a highly conserved timing mechanism, the circadian clock, which enables them to anticipate and thereby to adapt to these regular environmental challenges. This internal clock ticks under constant conditions with an approximately 24 hours period [1,2] and so relies on regular resetting by environmental signals indicative of the time of day (so-called *zeitgebers*) to ensure synchronization with the day-night cycle [3–5]. In turn, this timing mechanism directs circadian rhythmicity in almost all aspects of physiology and behaviour via a range of cell autonomous and systemic output signals. The importance of this endogenous timing mechanism is revealed by its high degree of conservation between organisms, ranging from cyanobacteria and fungi to humans, and the negative effects associated with its disruption. Sustained circadian clock disruption has been linked with an increased incidence of a range of diverse pathologies including cardiovascular and neurodegenerative disorders, cancer [6–8], diabetes [9], and obesity [10].

At the molecular level, the core of the circadian clock consists of a transcription-translation feedback loop (TTFL) [11]. In mammals, the CLOCK and BMAL proteins, members of the bHLH PAS family of transcription factors, heterodimerize and directly bind to the E-Box enhancer, thereby activating transcription of other clock genes, namely the *Period (per)* and *Cryptochrome* (*Cry*) genes which function as negative components within the clock mechanism. Following the CLOCK-BMAL-mediated transcriptional activation, the PER and CRY proteins accumulate in the cytoplasm and then translocate into the nucleus where they physically interact with the CLOCK-BMAL heterodimer and thereby suppress transactivation by this complex. Consequently, the expression levels of PER and CRY are decreased, leading to a reduction in the inhibitory effect on the CLOCK-BMAL complex and then below a critical threshold, allowing a new cycle to begin. This autoregulatory cycle takes around 24 hours to complete and is the driver of circadian rhythmicity [12]. In mammals, circadian clock regulation involves not only E-box mediated transcription control, but also the D-box enhancer which serves as the target for a clock-regulated output mechanism. D-box–driven transcriptional activation is mediated by a set of PAR (proline and acidic amino acid-rich) bZip transcription factors, comprising TEF (thyrotroph embryonic factor), HLF (hepatic leukemia factor) and DBP (D-box-binding protein). These factors selectively bind to D-box enhancer elements as both homo and heterodimers in the promoters of clock-controlled output genes [13–15]. The expression of PAR factor genes is regulated by the CLOCK-BMAL heterodimer through E-box mediated activation and thereby the D-box relays timing information from the core clock to the transcription of target genes [16]. Interestingly, over the course of vertebrate evolution the D-box has adapted to serve a very different function as part of the clock input pathway. Specifically, in fish species the D-box acts as a light responsive enhancer that directs light-induced transcription of certain key clock genes (specifically a subset of *per* and *cry* genes) as well as DNA repair genes. Via this mechanism the phase of the clock is adjusted to match the timing of the day-night cycle. Thus, fish possess directly light-entrainable peripheral clocks in contrast to the situation in mammals where light input to the circadian clock is indirectly mediated by specialized photoreceptors in the retina which entrain the master clock in the suprachiasmatic nucleus and thereby set the phase of peripheral tissue clocks via systemic signals [17,18].

Cryptochromes are members of the broader cryptochrome-photolyase family (CPF), a group of extensively conserved flavoproteins that harness blue light to perform many biological functions [19–23]. While cryptochromes serve as core circadian clock components as well as blue light photoreceptors [24], photolyases represent a highly conserved set of DNA damage repair enzymes which mediate photoreactivation repair. In photoreactivation the energy in visible light is harnessed to repair UV-induced DNA damage consisting of covalently crosslinked adjacent pyrimidine bases [25]. Three main classes of photolyase have been identified, the CPD photolyases (CPDphr) and 6–4 photolyases (6–4phr) which mediate the repair of CPD and 6–4 photoproducts respectively as well as the DASH photolyases (DASHphr) which repair UV-induced damage in the context of single stranded DNA [26–28]. While the CPF family is highly conserved during evolution, it has also changed significantly in certain species. For example, placental mammals and some blind cavefish lack functional photolyases and subsets of cryptochromes, suggesting that the cryptochrome and photolyase genes are subject to differential selective pressures. Given the high degree of structural similarity between cryptochromes and photolyases, it is tempting to speculate that there is overlap between the functions of these proteins. Interestingly, ectopic expression of marsupial CPDphr can restore circadian rhythmicity in the liver of CRY1/CRY2-deficient mice, suggesting that rhythmically expressed photolyases can substitute for CRY proteins in the mammalian circadian oscillator [29]. However, the degree of functional overlap between cryptochromes and photolyases remains poorly understood.

In this study we reveal significant overlap between cryptochrome and photolyase function in the context of the circadian clock in vertebrates. Fish models such as the zebrafish and medaka are ideally suited for studying this functionality due to their broad photic responsiveness. Specifically, they exhibit photoreactivation DNA repair as well as directly light entrainable circadian clocks in most cell types and even cell lines [30,31] where they also express a broad range of opsin photoreceptors, photolyases and cryptochromes [15]. We reveal that loss of 6–4phr results in abnormalities of rhythmic circadian clock gene expression in medaka cells. Furthermore, 6–4phr, but not CPDphr, represses Clock1-Bmal1-mediated transactivation via the E-box enhancer and enhances transcriptional activation mediated by Tef at the D-box enhancer. We demonstrate that photolyase domain of 6–4phr can physically interact with the D-box binding transcription factor Tefb and with multiple domains of the Clock1 and Bmal1 proteins, thereby interfering with heterodimerization of the Clock1-Bmal1 complex.

## Results

### Abnormal circadian clock gene expression upon loss of 6–4phr function

**Fig 1:**
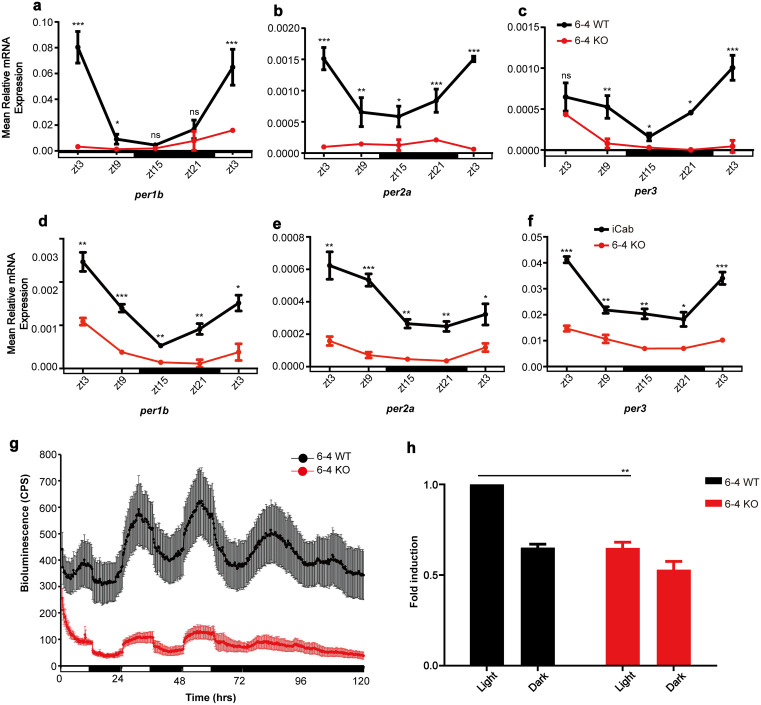
Circadian clock gene expression and regulation in loss of 6-4phr function medaka cells. **(a-f)** Medaka 6-4 wild type (WT) and mutant (KO) cell lines **(a-c)** and explanted wild type (iCab) and 6-4phr mutant (6-4 KO) fish fin clips **(d-f)** were incubated under light-dark cycle (LD) conditions (12 hours light and 12 hours dark) for 4 days to entrain their circadian clocks and thereafter sampled at regular timepoints through a complete LD cycle. The mRNA expression levels of the clock-regulated genes *per1b* and *per3* and the light-regulated gene *per2a* were analyzed by qRT-PCR. Relative mRNA expression calculated as mean ± SEM (n = 3) is plotted on the y-axes, while *zeitgeber* time (zt) sampling timepoints are plotted on the x-axes. **(g)** *In vivo* luciferase assay of medaka 6-4 WT (black trace) and KO (red trace) cell lines transfected with a luciferase reporter carrying the zebrafish clock-regulated *per1b* promoter. Bioluminescence (cps) values are plotted on the y-axis against time (hrs) on the x-axis. Each timepoint represents the mean ± SEM from independently transfected wells (n ≥ 8). White and black horizontal bars along the x-axis indicate the light and dark periods, respectively. **(h)** *In vitro* luciferase assay of medaka 6-4 WT (black) and KO (grey) cells transfected with a luciferase reporter construct containing 15 tandemly repeated copies of the D-box sequence derived from the zebrafish *cry1aa* gene promoter (*D-box*_*cry1aa*_*Luc*) and exposed to visible light or maintained in constant darkness. The fold induction of relative bioluminescence values is plotted on the y-axis against exposure conditions on the x-axis. The results are presented as mean ± SEM (n ≥ 3). A ß-galactosidase assay was employed for normalization of transfection efficiency. Significant thresholds are indicated by asterisks and p > 0.05, p < 0.05, p < 0.01, p < 0.001 are represented by ns, *, ** or ***, respectively. Three biological replicates were performed for each experiment.

It has been reported that marsupial CPDphr can functionally substitute for CRY proteins to restore a normal circadian clock in loss-of-function mouse mutants [29]. Is this functionality restricted to this artificial situation or does it reflect a broader natural photolyase function in vertebrates? To address this question, we analyzed CRISPR-Cas9 generated medaka cell lines carrying loss of function mutations in the *CPDphr*, *6–4phr* and *DASHphr* genes and their corresponding wild type (WT) controls [32]. All cell lines were cultured under light-dark cycle (LD) conditions (12 hours light and 12 hours dark) for several days to directly entrain their circadian clocks. Subsequently, we assayed the levels of various clock gene mRNAs in each line at regular time points to investigate whether there were any abnormalities in rhythmic expression. Specifically, the expression levels of a set of *period* genes (*per1b*, *per2a* and *per3*) were quantified at various timepoints throughout the light-dark cycle by qRT-PCR analysis. The results revealed low basal levels and arhythmic expression for all three *period* genes analyzed in the 6–4phr mutant cell line compared with the corresponding WT control cells (Fig 1a-c and Tab A in S1 Data). However, in the other mutant medaka cell lines (CPDphr and DASHphr mutants), rhythmic expression of these genes closely matched that of the wild type control cell line (S1 Fig and Tab F in S1 Data). Might this abnormality be restricted to these cell culture models? To confirm these initial *in vitro* results, we also measured the mRNA levels of these three *per* genes in cultured fin clips from the 6–4phr mutant fish as well as the corresponding WT controls (iCab) which were maintained under LD conditions for 4 days to entrain their circadian clocks. Consistent with the *in vitro* results, we observed low basal levels and arhythmic expression of *per* gene mRNA in the 6–4phr mutant compared with iCab fin clips (Fig 1d-f and Tab A in S1 Data). This reveals that among the three photolyase genes, loss of 6–4phr function has a greater impact on rhythmic clock gene expression in medaka.

To further investigate the molecular mechanism whereby 6–4phr influences the circadian rhythmicity of *per* gene expression we explored the possible role of 6–4phr in transcriptional regulation. Rhythmic transcription of *per1b* and *per3* is regulated directly by the circadian clock via E-box enhancer elements while the light-inducible expression of *per2a* relies on proximal E- and D-box elements [31,33,34]. Medaka 6–4phr mutant and wild type control cells were transiently transfected with a luciferase reporter driven by the zebrafish *per1b* promoter which includes multiple E-box enhancers [31]. Following transfection, cells were initially exposed to LD conditions in order to entrain their endogenous circadian clocks for 3 days followed by incubation under constant darkness for 2 days (free-running conditions). Consistent with our previous qRT-PCR analysis of endogenous *per1b* expression, we observed a substantial attenuation of clock-regulated rhythmic gene expression under both entraining and free-running conditions. These findings agree with 6–4phr playing a key role in E-box – mediated transcriptional control mechanisms in the medaka cell lines (Fig 1g and Tab A in S1 Data).

Light-driven transcription of the *per2a* gene has been demonstrated to be regulated by a D-box enhancer promoter element [35,36]. Therefore, we next tested the effect of loss of 6–4phr function on D-box enhancer-regulated transcription. Specifically, the 6–4phr mutant and WT cells were transfected with a heterologous reporter construct where 15 tandemly repeated copies of a D-box enhancer sequence derived from the *cry1aa* promoter, were cloned upstream of a minimal promoter which drives transcription of the luciferase reporter gene (*D-box*_*cry1aa*_*Luc*) [17]. Transfected cells were exposed to visible light for 15 hours while a control set of cells was maintained in constant darkness before preparing cell extracts and performing a luciferase assay. Our results revealed significant attenuation of light-induced luciferase expression in the 6–4phr mutant cells compared with WT control cells (Fig 1h and Tab A in S1 Data). Therefore, together these results are consistent with 6–4phr playing a regulatory role in both E-box and D-box directed transcription, contributing to both light- and clock-regulated gene expression.

### 6-4phr differentially modulates E-box and D-box binding transcription factors

**Fig 2:**
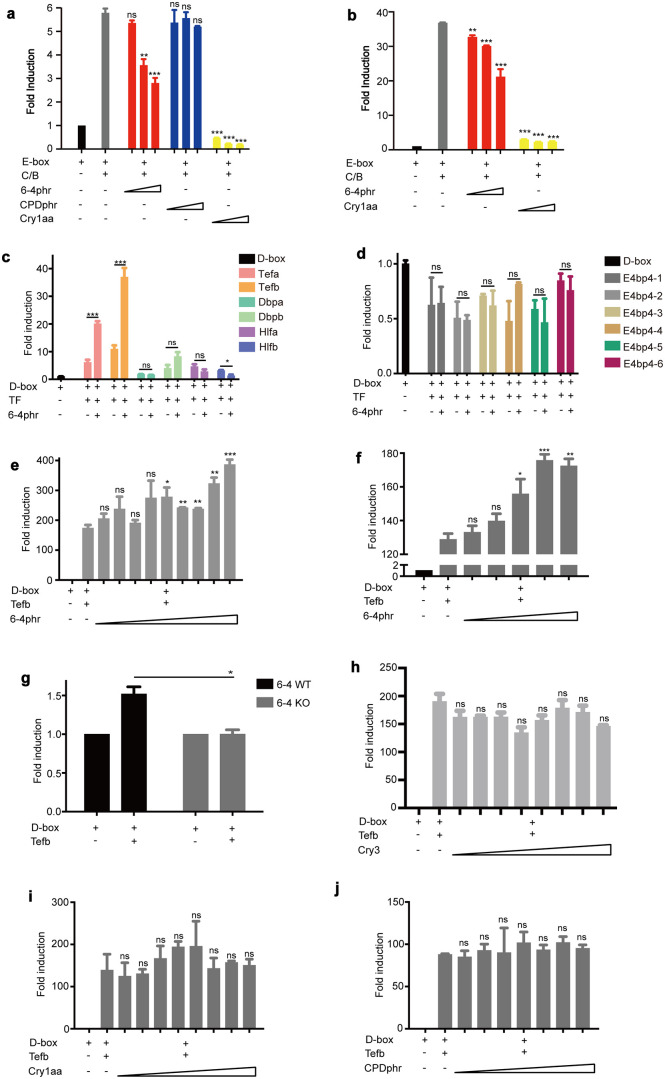
6–4phr differentially modulates E-box and D-box binding transcription factors. **(a-b)** *In vitro* luciferase assay of zebrafish PAC-2 **(a)** and mammalian 3T3 **(b)** cells co-transfected with an E-box luciferase reporter construct containing 4 tandemly multimerized copies of the E-box sequence derived from the zebrafish *per1b* gene promoter (*4xE-box (-7)*) and expression vectors for zebrafish Clock1a and Bmal1a, 6–4phr, CPDphr and Cry1aa (from 50 ng to 150 ng). **(c-d)** *In vitro* luciferase assay of zebrafish PAC-2 cells co-transfected with a D-box reporter construct containing 15 tandemly multimerized copies of the D-box sequence derived from the zebrafish *cry1aa* gene promoter (*D-box*_*cry1aa*_*Luc*) together with expression vectors for the six PAR factors, specifically Tefa, Tefb, Dbpa, Dbpb, Hlfa and Hlfb **(c)** and six E4bp4/nfil3 factors, specifically E4BP4–1 (nfil3-1a/e4bp4–1), E4bp4–2 (nfil3-2a/e4bp4–2), E4BP4–3 (nfil3-3b/e4bp4–3), E4BP4–4 (nfil3-1b.1/e4bp4–4), E4BP4–5 (nfil3-2b/e4bp4–5) and E4BP4–6 (nfil3-3a/e4bp4–6) **(d)** (25 ng) as well as zebrafish 6–4phr (30 ng). **(e-f, h-j)** *In vitro* luciferase assay of zebrafish PAC-2 **(e, h-j)** and mammalian 3T3 **(f)** cells co-transfected with the *D-box*_*cry1aa*_*Luc* reporter construct and expression vectors for zebrafish Tefb (25 ng) together with the titration of expression vectors for 6–4phr **(e-f)**, Cry3 **(h)**, Cry1aa **(i)** or CPDphr **(j)**. **(g)** *In vitro* luciferase assay of medaka 6–4 WT and KO cell lines co-transfected with the *D-box*_*cry1aa*_*Luc* reporter construct and the expression vector for zebrafish Tefb. The expression plasmids included in each transfection are indicated below each graph. Fold induction of relative bioluminescence values is plotted on the y-axis. The results are indicated as mean ± SEM (n = 3). A ß-galactosidase assay was used to standardize for transfection efficiency. Three biological replicates were performed for each experiment. Significant thresholds are denoted by asterisks and p > 0.05, p < 0.05, p < 0.01, p < 0.001 are represented by ns, *, ** or ***, respectively.

As a part of the central TTFL in the molecular circadian clock mechanism, at a critical stage of the circadian cycle, the Per and Cry proteins suppress transactivation by the Clock-Bmal heterodimer. Given the overall structural similarity between photolyases and cryptochromes, we speculated that 6–4phr may potentially function as a negative regulator of Clock-Bmal-mediated transactivation. In contrast, based on our qRT-PCR results (S1a-c Fig and Tab F in S1 Data), although CPDphr shares a similar structure with 6–4phr, this enzyme should not be expected to influence Clock-Bmal function. To test this hypothesis, wild type zebrafish cells (PAC-2 cells) were co-transfected with a luciferase reporter under the transcriptional control of a minimal promoter carrying 4 tandemly repeated copies of a canonical E-box element derived from the zebrafish *per1b* promoter (*4xE-box (-7)* [31]) together with expression vectors encoding zebrafish Clock1a and Bmal1a. These constructs were either co-transfected with expression vectors for 6–4phr, CPDphr or a Cry1aa expression vector used as an experimental positive control. While confirming that Cry1aa serves as a potent repressor of the transactivating function of Clock1a-Bmal1a on the E-box reporter, importantly our results also revealed that 6–4phr co-expression led to a dose-dependent suppression of Clock1a-Bmal1a-mediated activation. In contrast, co-expression with CPDphr failed to elicit any significant repression of Clock1a-Bmal1a transactivation (Fig 2a and Tab B in S1 Data). Comparable results were obtained by transfecting the same constructs into the murine 3T3 cell line which lacks endogenous photolyase genes (Fig 2b and Tab B in S1 Data). Therefore, these findings are consistent with 6–4phr, but not CPDphr, sharing a Clock-Bmal inhibitory function with Crys, resulting in down regulation of E-box-mediated transcription.

We next wished to investigate how 6–4phr influences D-box enhancer-binding transcription factor function. Six PAR bZip transcription factors (Tefa, Tefb, Hlfa, Hlfb, Dbpa and Dbpb) and seven E4bp4/nfil3 family members (nfil3-1a/e4bp4–1, nfil3-2a/e4bp4–2, nfil3-3b/e4bp4–3, nfil3-1b.1/e4bp4–4, nfil3-2b/e4bp4–5, nfil3-3a/e4bp4–6 and nfil3-1b.2) [13,14] are recognized as D-box binding bZip transcription factors in fish. We initially investigated whether ectopic expression of 6–4phr influences transcriptional regulation by twelve of these transcription factors in a co-transfection assay in PAC-2 cells. Specifically, the *D-box*_*cry1aa*_*Luc* reporter was co-transfected together with expression vectors for each of the twelve D-box – binding transcription factors in the presence or absence of the *6–4phr* expression vector*.* While co-transfection with expression vectors for all six PAR bZip factors resulted in an increase in bioluminescence derived from the D-box reporter construct, co-expression of 6–4phr together with tefa, tefb and dbpb resulted in higher levels of bioluminescence than with the PAR bZip factors alone (Fig 2c and Tab B in S1 Data). However, in the case of co-expression of the E4bp4/nfil3 factors with the D-box reporter, levels of bioluminescence remained relatively low in the presence or absence of the 6–4phr expression vector (Fig 2d and Tab B in S1 Data).

To validate these initial results, we focused on the inducing effect of 6–4phr on D-box – mediated transcriptional activation by Tefb, the factor displaying the strongest activation in our assay. We co-transfected the *D-box*_*cry1aa*_*Luc* reporter with a fixed amount of Tefb and increasing amounts of the 6–4phr expression vectors. We observed a dose-dependent increase in the Tefb, D-box – mediated transactivation (Fig 2e and Tab B in S1 Data). This result was also confirmed in mouse 3T3 cells where we observed a comparable, dose-dependent increase in Tefb-induced activation of D-box reporter transcription in the presence of increasing amounts of co-transfected 6–4phr expression vector (Fig 2f and Tab B in S1 Data).

As an alternative test for the contribution of endogenous 6–4phr to Tefb regulation, we co-transfected the *D-box*_*cry1aa*_*Luc* reporter construct and Tefb expression vector into the medaka 6–4phr mutant and WT cell lines. Consistent with the previous results obtained using exogenously expressed 6–4phr, lower levels of Tefb-induced D-box reporter expression were observed in the 6–4phr mutant cells compared with the WT cells (Fig 2g and Tab B in S1 Data).

How specific are the effects of 6–4phr on Tefb regulation? Do Crys or other photolyases also influence activation by this PAR bZip factor? A comparable experiment was performed by co-transfecting PAC-2 cells with the *D-box*_*cry1aa*_*Luc* reporter, a fixed amount of Tefb expression construct and increasing levels of Cry3, Cry1aa or CPDphr expression vectors. Based on phylogenetic analysis, fish Cry3 represents a close relative of 6–4phr while Cry1aa bears more similarity with mammalian cryptochromes [37]. Instead, CPDphr belongs to a distinct group of photolyases catalyzing the repair of CPD photoproducts [19]. In all cases, we failed to observe any significant effect on Tefb activation of D-box reporter transcription (Fig 2h and 2j and Tab B in S1 Data).

### 6-4phr physically interacts with multiple circadian clock transcription factors

**Fig 3:**
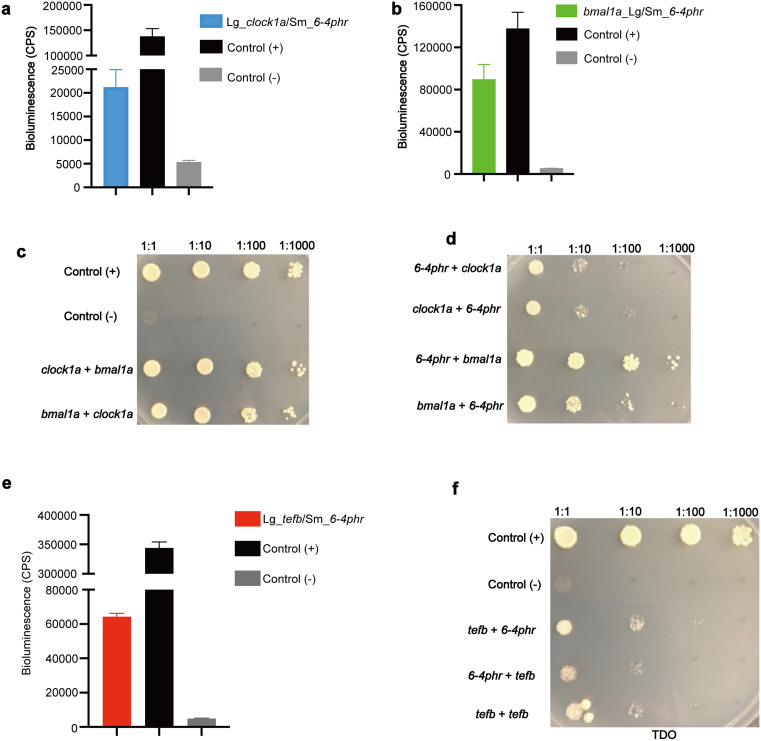
6–4phr physically interacts with circadian clock transcription factors. **(a-b, e)** NanoLuc Binary Technology (NanoBiT) assay to test the physical interaction between zebrafish Clock1a **(a)**, Bmal1a **(b)** or Tefb **(e)** and 6–4phr. The full-length coding sequences of *clock1a*, *bmal1a*, *tefb* and *6–4phr* were cloned into pcDNA 3.1 or the pCS2-MTK vector incorporating a CMV promoter and fused either with LgBiT (at the 5’ end for *clock1a* and *tefb* or 3’ end for *bmal1a*) or with SmBiT (at the 3’ end for *6–4phr*). The PRKACA:PRKAR2A pair was used as a constitutive positive control, while the Halo Tag-SmBiT was used as a negative control. Bioluminescence (cps) values are plotted on the y-axes with the mean ± SEM (n ≥ 3). **(c-d, f)** Yeast 2 hybrid assay (Y2H) to test the physical interaction between zebrafish Clock1a, Bmal1a **(c-d)** or Tefb **(f)** with 6–4phr. The full-length coding sequences of *clock1a*, *bmal1a*, *tefb* and *6–4phr* were cloned into the pGBKT7 or pGADT7 vectors and the DNA-binding domain of GAL4 (BD) or activation domain of GAL4 (AD) was fused at the N- or C-terminus. The targeted constructs were transformed separately into the yeast strains AH109 or Y187 and the yeast transformants were patched onto agar plates including synthetic medium without leucine, tryptophan and histidine (TDO). A titrated dilution was performed to show the interaction between each pair of proteins. Three biological replicates were performed for each experiment.

Protein-protein interactions are essential for the core timing function of the circadian clock. Therefore, do the effects of 6–4phr on transcriptional regulation by the E-box and D-box result from a direct interaction between 6–4phr and the transcription factors that bind to them? As a first step to test for a direct physical interaction between 6–4phr and the Clock-Bmal heterodimer, we performed a NanoBiT (NanoLuc Binary Technology) - based assay. Expression constructs for the three proteins linked at the N- or C-terminus with the large (LgBiT) or the small (SmBiT) subunits of the Nano-luciferase enzyme were prepared. Expression of the various NanoBiT fusion constructs was initially confirmed by transfection into PAC-2 cells followed by western blotting with an anti-LgBiT or epitope-specific anti-Myc tag antibody (S2a-c Fig and Tab G in S1 Data). As a positive control, Clock1a and Bmal1a NanoBiT expression constructs were co-transfected into PAC-2 cells. We observed a robust induction of furimazine-derived bioluminescence, consistent with the well-documented heterodimerization of these two bHLH-PAS domain transcription factors (S2d Fig and Tab G in S1 Data). Subsequently, 6–4phr and Clock1a or Bmal1a NanoBiT constructs were co-transfected into PAC-2 cells in various pairwise combinations and NanoBiT luciferase-catalyzed bioluminescence was assayed. Our results reveal that co-expression of Clock1a fused with the LgBiT subunit at the N-terminus and 6–4phr linked with SmBiT at the N-terminus resulted in elevated bioluminescence signals (Fig 3a and Tab C in S1 Data). Furthermore, the Bmal1a protein fused with LgBiT at the C-terminus and 6–4phr fused with SmBiT at the N-terminus also showed significantly elevated bioluminescence values (Fig 3b and Tab C in S1 Data). These results are consistent with both Clock1a and Bmal1a proteins physically interacting with 6–4phr.

This result was confirmed with a yeast two hybrid protein-protein interaction assay. Specifically, full-length *6–4phr*, *clock1a* and *bmal1a* coding sequences were fused to the coding sequences for the GAL4 DNA-binding domain (BD) or the GAL4 activation domain (AD) and then the expression vectors were transformed into yeast cells. For selection of constructs, agar plates including synthetic media devoid of leucine and tryptophan (LW) were employed with various dilutions of each combination (S2e and S2f Fig) and growth on media lacking leucine, tryptophan and histidine (TDO) was tested in order to evaluate the interactions. The various dilutions were used to test the interaction strength between each combination. Consistent with our NanoBiT assay results, the colony growth confirms that Clock1a and Bmal1a display a strong physical interaction, and also that both Clock1a and Bmal1a interact robustly with 6–4phr (Fig 3c and 3d).

Does the induction of Tefb mediated transcription from the D-box enhancer by 6–4phr also rely upon direct interaction between the two factors? To address this question, initially several Tefb NanoBiT fusion constructs were generated and co-transfected in various pairwise combinations into PAC-2 cells. Consistent with the formation of Tefb-Tefb homodimers via dimerization of the bZip domains, we observed significantly elevated bioluminescence signals upon transfection with certain pairwise combinations of the Tefb fusion constructs (S2g Fig and Tab G in S1 Data). Co-transfection of PAC-2 cells with the Tefb and 6–4phr NanoBiT constructs also revealed significantly elevated bioluminescence levels, indicating that 6–4phr has the capacity to physically interact with the Tefb protein (Fig 3e and Tab C in S1 Data). We again validated these results using the yeast two hybrid assay. Expression vectors carrying the Tefb coding sequence fused to the GAL4 DNA-binding domain (BD) or the GAL4 activation domain (AD) were generated and then transformed into AH109 and Y187 yeast cells, respectively, and mated with corresponding 6–4phr expressing yeast strains. The colony growth results were used to test the expression (S2h Fig) and confirm that 6–4phr can physically interact with the Tefb protein (Fig 3f).

### Photolyase domain of 6–4phr is responsible for the interaction with Clock1a, Bmal1a and Tefb

**Fig 4:**
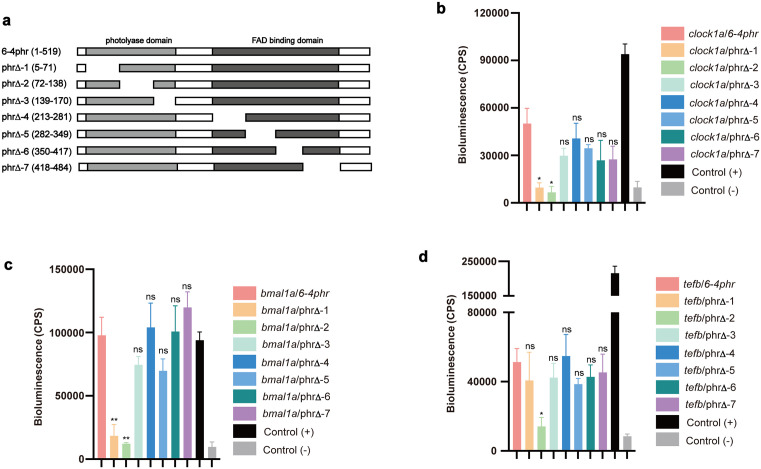
The photolyase domain of 6-4phr interacts with Clock1a, Bmal1a and Tefb. **(a)** Schematic representation of the set of N-terminally, SmBiT-tagged 6-4phr sub-deletion constructs used to test the regions of the 6-4phr protein mediating interaction with full length Clock1a, Bmal1a and Tefb. **(b-d)** NanoBiT assays testing which domains of 6-4phr mediate the interactions with the Clock1a **(b)**, Bmal1a **(c)** or Tefb **(d)** proteins. SmBiT-tagged 6-4phr sub-deletions as well as the LgBiT - tagged full length Clock1a, Bmal1a or Tefb expression constructs were cotransfected into zebrafish PAC-2 cells. The bioluminescence assay results (cps) are plotted on the y-axes as means ± SEM (n ≥ 3). Three biological replicates were performed for each experiment. Significant thresholds are indicated by asterisks and p > 0.05, p < 0.05, p < 0.01, p < 0.001 are represented by ns, *, ** or ***, respectively.

The 6–4phr protein possesses two highly conserved domains involved in chromophore binding. A FAD binding domain which binds flavin adenine dinucleotide and a photolyase domain which binds chromophores such as 5,10-Methenyltetrahydrofolate (MTHF). Via these chromophores, primarily FAD, the 6–4phr protein harvests light energy to catalyse photoreactivation repair of UV-induced 6–4 photoproducts [26,27]. Are these domains crucial for the interaction of 6–4phr with circadian clock components? To address this question, we generated several sub-deletions of 6–4phr fused with SmBiT (phr∆-1–7, Fig 4a) and then tested the interaction of these deletion constructs with full-length Clock1a, Bmal1a or Tefb fused to LgBiT. The expression of these sub-deletions was initially validated in transfected zebrafish PAC-2 cells by western blotting using an epitope-specific anti-Myc tag antibody (S3a and S3b Fig and Tab H in S1 Data). Each 6–4phr sub-deletion expression vector was then co-transfected in PAC-2 cells together with the expression constructs for LgBiT-tagged, full-length Clock1a, Bmal1a or Tefb. Cotransfection with an expression vector for SmBiT-tagged, full length 6–4phr served as a positive control. Bioluminescence assay results revealed that the phr∆-1 and phr∆-2 deletions disrupted interaction with the Clock1a and Bmal1a proteins, while only the phr∆-2 deletion interrupted the interaction with the Tefb protein. These findings are consistent with the region of the 6–4phr protein which physically interacts with these circadian clock components (Fig 4b-d and Tab D in S1 Data), overlapping with the photolyase domain.

### Multiple functional domains of Clock1a and Bmal1a are responsible for the interaction with 6–4phr

**Fig 5:**
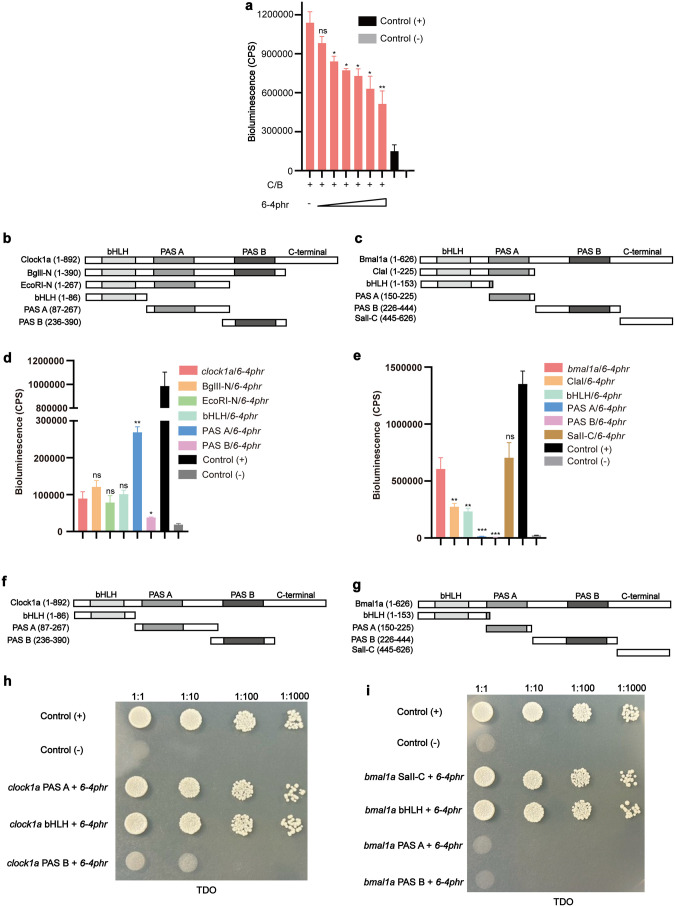
Multiple functional domains of Clock1a and Bmal1a are responsible for the interaction with 6-4phr. **(a)** NanoBiT assay to test the effect of 6-4phr on Clock1a-Bmal1a heterodimerization. The LgBiT-tagged zebrafish full length Clock1a and Bmal1a expression constructs were transfected into PAC-2 cells at optimized concentrations together with a titration of expression vector for the untagged 6-4phr. **(b-c)** Schematic representation of the N terminally, LgBiT-tagged Clock1a **(b)** and C terminally, LgBiT-tagged Bmal1a **(c)** deletion constructs used to test for interactions with full length 6-4phr. **(d-e)** NanoBiT assay to test the domains of Clock1a **(d)** and Bmal1a **(e)** responsible for the interaction with 6-4phr. The LgBiT-tagged_Clock1a or_Bmal1a_deletion expression vectors together with the SmBiT-tagged full length 6-4phr expression vector were transfected into zebrafish PAC-2 cells. Bioluminescence (cps) values are plotted on the y-axes as mean values ± SEM (n = 3). **(f-g)** Schematic representation of Clock1a **(f)** and Bmal1a **(g)** deletion mutants fused to the GAL4 activation domain (AD) or GAL4 DNA binding domain (BD) and used to test for interactions with full length 6-4phr fused to the GAL4 DNA binding domain (BD) or GAL4 activation domain (AD). **(h-i)** Yeast 2 hybrid assay to test the critical domains of Clock1a **(h)** and Bmal1a **(i)** responsible for their interaction with 6-4phr. Clock1a and Bmal1a deletions and full length 6-4phr were transformed separately into the yeast strains AH109 or Y187 and the yeast transformants were patched onto agar plates including synthetic medium without leucine, tryptophan and histidine (TDO). A titrated dilution was performed to show the interaction between each combination. Three biological replicates were performed for each experiment. Significant thresholds are denoted by asterisks and p > 0.05, p < 0.05, p < 0.01, p < 0.001 are represented by ns, *, ** or ***, respectively.

The arhythmic *period* gene expression observed in 6–4phr mutant cells and tissues points to interactions between 6–4phr, Clock1a and Bmal1a proteins playing a key role in the core clock TTFL mechanism. We therefore explored in more detail how 6–4phr interacts with Clock1a and Bmal1a. Does 6–4phr repress the transactivation of the Clock1a-Bmal1a heterodimer directly by interfering with their heterodimerization, by influencing their interaction with other transcriptional co-activators or with the RNA polymerase machinery itself [38,39]? To address these questions, we performed a NanoBiT assay involving co-transfection of PAC-2 cells with fixed amounts of the Clock1a and Bmal1a NanoBiT fusion expression constructs together with increasing amounts of an expression vector for the untagged 6–4phr. The results reveal a significant decrease in the interaction between the Clock1a and Bmal1a NanoBiT fusion proteins in the presence of increasing amounts of co-transfected 6–4phr (Fig 5a and Tab E in S1 Data). This data is consistent with 6–4phr repressing Clock1a-Bmal1a-activated transcription by interfering with the formation of Clock1a-Bmal1a heterodimers.

Which domains of Clock1a and Bmal1a are essential for the physical interaction with 6–4phr? To address this question, multiple sub-deletion constructs based on the Clock1a and Bmal1a NanoBiT constructs were generated in order to systematically eliminate each functional domain of the Clock1a and Bmal1a proteins (Fig 5b and 5c) and their expression was confirmed by western blotting (S3c-g Fig and Tab H in S1 Data). Zebrafish PAC-2 cells were then co-transfected with each of these deletion constructs together with the construct expressing full-length 6–4phr fused with SmBiT at the N-terminus. The NanoBiT results were then validated with a yeast two hybrid assay where various portions of Clock1a and Bmal1a were fused to the GAL4 DNA-binding domain (BD) or the GAL4 activation domain (AD) (Fig 5f and 5g). Then these constructs were transformed into yeast cells for mating with cells transformed with the full-length 6–4phr in order to test for protein-protein interaction (S3h and S3i Fig). Together, these results revealed that the integrity of the bHLH and PAS A domains of Clock1a as well as the bHLH and C-terminal domains of Bmal1a is essential for the physical interaction with the 6–4phr protein (Fig 5d, 5e, 5h and 5i and Tab E in S1 Data).

## Discussion

**Fig 6:**
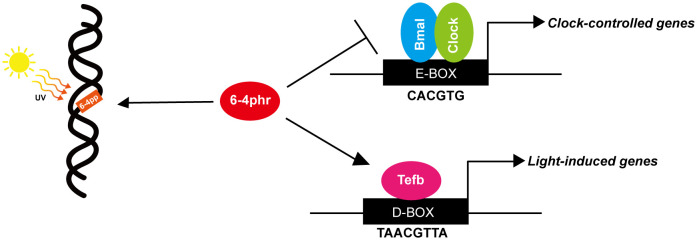
Schematic representation of 6-4phr functionality. The Clock-Bmal heterodimer can activate the transcription of clock-controlled-genes by binding to the E-box enhancer and this transactivation can be repressed by Per and Cry. We reveal that 6-4phr which repairs UV-induced 6-4 photoproducts via photoreactivation, may also be involved in transcriptional regulation within the circadian clock molecular mechanism. Specifically, 6-4phr is able to repress transactivation mediated by the Clock-Bmal complex at the E-box enhancer via binding to the bHLH domain of Clock and Bmal and interfering with heterodimerization. Furthermore, 6-4phr also positively regulates D-box mediated transcriptional control of light-induced-genes by physical interaction with the Tefb protein.

Photolyases are highly conserved from bacteria to higher vertebrates with the notable exception of the placental mammals which lack photolyase genes. Our results have revealed that in fish, the 6–4phr protein which is well documented to mediate photoreactivation repair of UV-induced 6–4 photoproducts in DNA is also involved in the regulation of the circadian clock. We demonstrate that loss of 6–4phr function leads to loss of rhythmic expression of *period* clock genes in cultured medaka cells and fin clips. 6–4phr downregulates transcriptional activation by Clock1a-Bmal1a via interfering with heterodimerization. Furthermore, the photolyase domain of 6–4phr together with the bHLH and PAS A domains of Clock1a as well as the bHLH and C-terminal domains of Bmal1a mediate physical interaction between 6–4phr and these core clock proteins. Interestingly, 6–4phr can also influence transcriptional regulation via a distinct enhancer element, the D-box. We reveal that the photolyase domain of 6–4phr can physically interact with the PAR bZip transcription factor Tefb which binds to the D-box enhancer and thereby increases transcriptional activation. In contrast, loss of CPD photolyase function does not significantly affect circadian clock function or modulate E-box and D-box regulated transcription. These findings point to dual functionality of 6–4phr (Fig 6).

Our results support previous evidence that members of the CPF family can exhibit dual functionality. For example, ectopic expression of the marsupial *Potorous tridactylus* PtCPD can substitute for CRY function in the mouse circadian oscillator [29]. Furthermore, in the marine diatom, *Phaeodactylum tricornutum*, a member of the CPF family named PtCPF1, displays DNA repair activity for 6–4 photoproducts, and meanwhile, also suppresses transactivation of the Clock-Bmal heterodimer in the context of the circadian clock [40]. Moreover, in the green alga, *Ostreococcus tauri*, a member of the CPF family, termed OtCPF1 also exhibit dual functions. Specifically, OtCPF1 has the capacity to repair 6–4 photoproducts, and is also involved in circadian clock regulation by mediating Clock-Bmal-activated transcription at the E-box enhancer [41]. In addition, as well as serving as core clock components, human CRYs have also been implicated in monitoring UV-induced DNA damage [42]. The structure and function relationship of both cryptochromes and photolyases have been extensively studied in relation to interaction with Clock-Bmal and the catalysis of DNA repair respectively. However, the mechanisms whereby photolyases also interact with the circadian clock transcriptional machinery remain unexplored. Photolyases and cryptochromes both share a highly conserved photolyase domain as well as FAD binding domain, which are crucial for the interaction with chromophores and the harvesting of light energy. However, our results specifically point to some degree of dual functionality in the case of the 6–4phr photolyase domain which also appears to be involved in mediating protein-protein interactions with a set of light- and clock-regulated transcription factors. Therefore, photolyases and cryptochromes both display the structural features of blue light photoreceptors as well as transcriptional cofactors [19–23]. The animal and plant cryptochromes are not close relatives [21] and from phylogenetic analysis, plant cryptochromes exhibit a higher sequence similarity with CPDphr [43], while animal cryptochromes are more closely related to 6–4phr [44,45], which is consistent with our findings revealing the specificity of transcriptional regulation by vertebrate 6–4phr within the circadian clock.

Clues as to the selective pressures that have shaped the evolution of the CPF family come from vertebrate groups. Interestingly, placental mammals as well as certain species of blind cavefish lack functional photolyase genes [46,47]. The loss of photoreactivation DNA repair over the course of blind cavefish evolution, completely shielded from the effects of sunlight exposure would appear to be a logical outcome. However, the absence of photolyase genes in the placental mammal lineage is more difficult to account for. These observations have led to a theory that during evolution, the ancestors of placental mammals experienced a “nocturnal bottleneck” [48,49]. Specifically, it is predicted that as part of a strategy to avoid predation from diurnal carnivorous dinosaurs, these mammalian ancestors adopted a subterranean, nocturnal lifestyle that ultimately led to a loss of sunlight-dependent functions, including photoreactivation DNA repair. Nevertheless, many lines of evidence point to the conservation of circadian clock function still being essential in subterranean environments to efficiently exploit limited food availability [50–53]. This may well account for the conservation of cryptochromes in mammals that serve as core clock components. Furthermore, while birds, reptiles and amphibia possess multiple cryptochromes that do not serve as repressors of Clock and Bmal [54–56], these additional cryptochromes are also not encountered in the mammalian lineage. These observations indicate that light exposure may have played a fundamental role in shaping the evolution of the CPF family of flavoproteins.

Previous studies have documented how cryptochromes interact with CLOCK and BMAL. CRY can tightly interact with the C-terminus of BMAL [57,58] and thereby potently repress the activity of the CLOCK-BMAL heterodimer in mammals. However, in zebrafish, Cry1aa physically interacts with the PAS B domain of Clock and multiple domains of Bmal, including the bHLH, PAS B and C-terminal domains to interfere with the formation of the Clock-Bmal heterodimer [59]. This suggests some degree of species-specificity in the way that Cry interacts with Clock and Bmal. Consistently, here we reveal that the interaction between 6–4phr and Clock1a or Bmal1a relies on the bHLH and PAS A domains of Clock1a and the bHLH and C-terminal domains of Bmal1a which differs from the signatures of interaction with Cry1aa.

We reveal that 6–4phr can also interact with Tefb which regulates transcription via the D-box enhancer, however, contrary to the downregulatory effects of 6–4phr on the E-box, in this case the photolyase acts to upregulate transcriptional activation. In Arabidopsis, CRY2 cooperates with a transcriptional factor, CIB1 (cryptochrome-interacting basic helix-loop-helix 1), to regulate transcription by enhancing transcriptional activity of CIB1 under blue light [60]. In addition, CRY in *Drosophila* has the capacity to bind to TIM (Timeless), thereby resulting in proteolysis which reduces TIM levels and resets the phase of the circadian clock in a light-dependent way [61]. Our findings documenting transcriptional regulation by 6–4phr at the D-box enhancer provides important new insight into the diverse functionality of CPF members in vertebrates.

The catalysis of DNA repair by the photolyase proteins is closely related to their property of harvesting light. Our results which reveal a circadian clock function for 6–4phr further broadens the functional links of this protein with light. By suppressing transcriptional activation by Clock and Bmal, 6–4phr seems to act in an analogous fashion to cryptochromes. In addition, like cryptochrome genes such as *cry1aa*, transcription of this photolyase gene is strongly activated by light exposure via D-box enhancer promoter elements [35,36]. Light is the principal zeitgeber for the clock, hence potentially light-induced expression of *6–4phr* may play a vital part in resetting the clock’s phase in response to the ambient day-night cycle. Furthermore, given the capacity of 6–4phr to elevate Tefb transcriptional activation of the D-box, it is tempting to speculate that this photolyase may serve as an element in a feedback mechanism that adapts the transcriptome to light exposure. Bioinformatic studies as well as systematic, unbiased promoter analysis in zebrafish cells has demonstrated that several photolyase and cryptochrome genes possess conserved D-box enhancers involved in transcriptional mediation by light, and canonical E-box enhancer elements acting as circadian clock-regulated enhancer elements [33,36,47]. For all these reasons, it is evident that photo-activated DNA repair and photo-entrained circadian rhythmicity represent tightly interconnected functions that have coevolved within the cryptochrome and photolyase gene families.

## Materials and methods

### Fish maintenance and dissection of tissues

Wild type (iCab) and 6–4phr mutant (6–4 KO) medaka (*Oryzias latipes*) fish were maintained in the fish facility of the Institute of Biological and Chemical Systems, Biological Information Processing (IBCS-BIP) at the Karlsruhe Institute for Technology (KIT). The 6–4phr mutant fish line was generated by CRISPR-Cas9-based mutagenesis at Osaka University, Japan as previously described [32]. The fish were anesthetized using 0.02% Tricaine (Sigma Aldrich) diluted in embryo rearing medium (ERM) for a few minutes and thereafter fin clips were excised using a scalpel and placed in culture. The fish were then transferred to fresh ERM medium for recovery. The experiments performed with fish were conducted in compliance with the European Legislation for the Protection of Animals used for Scientific Purposes (Directive 2010/63/EU) (General license for fish maintenance and breeding: Az.: 35-9185.64/ BH KIT IBCS-BIP, Karlsruhe Institute of Technology (KIT)) and adhered to the animal protection standards of Germany.

### Explanted fin clip cultures, cell culture and transfection

iCab and 6–4 KO fish fin clips were excised using a scalpel, washed three times in 1 X PBS and Leibovitz’s L15 medium (Gibco) and then cultured in Leibovitz’s L15 medium supplemented with Fetal Bovine Serum (FBS) (Gibco) and appropriate antibiotics (Gibco). A cell line derived from zebrafish embryos (PAC-2) as well as the wild type and mutant medaka cell lines generated from medaka embryos (6–4/CPD/DASH WT/KO) were maintained in Leibovitz’s L-15 medium´ supplemented with Fetal Bovine Serum (FBS) and appropriate antibiotics. All these cell lines were cultured at 26°C and transfected according to the manufacturer’s instructions using FuGene HD transfection reagent (Promega) with a ratio of FuGene (μl) to plasmid DNA (μg) of 4:1.

### Gene expression analysis

Medaka wild type and photolyase mutant cells as well as fin clips were plated in petri dishes and maintained under LD cycle (12 hours light and 12 hours dark) conditions for 4 days. Afterwards, Trizol Reagent (Invitrogen) was added to the fin clips and cell layers then the lysates were transferred to 1.5 ml Eppendorf tubes and frozen at -80°C. Fin clips as well as cell samples were disrupted and homogenized by syringing repeatedly through a 25 Guage needle. Chloroform was added to separate RNA from the DNA and protein mixture and isopropanol was employed for RNA precipitation by following the manufacturer’s instructions. The integrity and quantity of the ribosomal 28S, 18S and 5S RNA bands was visualized by electrophoresis on a 1% agarose gel.

1 μg of total RNA was used for synthesis of cDNA and subsequently digested with RQ1 DNase co-incubated with a RNase Inhibitor. RQ1 DNase stop solution and a random primer were added to the mixture, and then a master mix (RT Buffer + dNTPs + RevertAid RT Enzyme) was prepared and added to the cDNA mixture and incubated according to the manufacturer’s instructions (Thermo Fisher). Electrophoresis through a 1% agarose gel was used to verify the quality of quantity of synthesized cDNA.

Quantitative Real-time PCR (qRT-PCR) was performed using the StepOnePlus Real-Time PCR System (Thermo Fisher) according to the manufacturer’s instructions. Medaka mitochondrial ribosomal protein S18B (*MRPS18B*) mRNA expression was used for normalization to calculate the relative mRNA expression levels of clock genes by the 2^-ΔΔCT^ method. A standard curve was plotted to test the primer efficiency for amplifying the *per* gene transcripts. The primer sequences used and test results are listed in S1 Table.

### Plasmid cloning

Several plasmids (*per1b-Luc* [31], *D-box*_*cry1aa*_*Luc* [17], *4xE-box(-7)* [31], and pCS2-MTK expression constructs for zebrafish *clock1a*, *bmal1a*, *6–4phr*, *CPDphr* [47], *cry1aa* as well as *tefa, tefb, dbpa, dbpb, hlfa, hlfb, nfil3-1a/e4bp4–1, nfil3-2a/e4bp4–2, nfil3-3b/e4bp4–3, nfil3-1b.1/e4bp4–4, nfil3-2b/e4bp4–5 and nfil3-3a/e4bp4–6* [13]) were maintained using standard methods in the Foulkes lab’s plasmid collection.

The full-length NanoBiT constructs containing *clock1a*, *bmal1a*, *6–4phr* and *tefb* coding sequences fused to coding sequences for the LgBiT or SmBiT domains at the N- or C-termini were generated by a PCR-based cloning strategy. Specifically, the inserts were PCR amplified using Q5 High-Fidelity DNA polymerase (New England Biolabs) and ligated with the pCS2-MTK or pcDNA3.1 vectors by T4 ligase (New England Biolabs). All of the NanoBiT Clock1a, Bmal1a and 6–4phr subdeletion expression plasmids were generated by using the Q5 Site-Directed Mutagenesis Kit (New England Biolabs) according to the manufacturer’s instructions. The primers used for construction are listed in S2 Table.

All the yeast two-hybrid construct cloning inserts including *clock1a* and its subdeletions, *bmal1a* and its subdeletions, *6–4phr* and *tefb* as well as the pGBKT7, pGADT7 and pcDNA3.1 cloning vectors were PCR amplified using Q5 High-Fidelity DNA polymerase (New England Biolabs). Plasmid fragments were assembled by employing the NEBuilder HiFi DNA Assembly Cloning Kit (New England Biolabs). All the insert fragments incorporated 25 bp of overlapping sequences relative to the neighboring vector fragment. Standard transformation protocols for *Escherichia coli* were used. The identity of all freshly generated and re-transformed plasmids was confirmed by appropriate restriction enzyme (New England Biolabs) digestion of mini preparation (QIAGEN) samples and sequencing of midi or maxi preparation (QIAGEN) samples by a commercial supplier (Microsynth Seq Lab). A NanoDrop Spectrophotometer (PeqLab) was used to assess the concentration and purity of all plasmid preparations. All the primers used for yeast two hybrid assay plasmid construction and mutagenesis are presented in S3 Table.

### *In vitro* luciferase assay

Cells were seeded in a 24-well plate and the next day were transfected with various plasmids of interest by using FuGene HD transfection reagent (Promega). A ß-galactosidase expression vector (pcDNA3.1/Myc-His/lacZ, Invitrogen) was included in each transfected sample to control for transfection efficiency and the final DNA quantity was adjusted by the addition of varying amounts of empty pCS2-MTK or pcDNA 3.1 vectors to ensure equal amounts of total DNA were included in each transfection. Cells were lysed by addition of firefly luciferase lysis buffer (0.1 M Tris acetate (pH = 7.5), 2 mM EDTA, 1% Triton X 100) and then collected and stored at -80°C until used for *in vitro* luciferase and ß-galactosidase assays.

I*n vitro* luciferase assays were performed using the Luciferase Assay System kit (Promega) according to the manufacturer’s instructions and bioluminescence levels were assayed using a Victor Light Luminescence Plate Reader (Perkin Elmer). The ß-galactosidase assay was performed using a standard colorimetric assay and optical densities were measured using a SpectraMax iD3 Microplate Reader at a wavelength of 420 nm.

### *In vivo* luciferase assay

Cells were seeded in a white 96-well plate (Perkin Elmer) and transfected with different plasmids of interest using FuGene HD transfection reagent (Promega) according to the manufacturer’s instructions. Thereafter, a beetle luciferin potassium salt solution (200 mM) (Promega) was added to the culture medium in each well (luciferin:medium = 1:250) and the plate was sealed with plastic sealing foil. The plates were then transferred to a Topcount NXT automatic scintillation counter (Perkin Elmer) under different lighting conditions (light/dark cycle or constant darkness). The data was analyzed by the I-and-A Excel plug-in (S. Kay, Scripps Research Institute).

### Western blotting analysis

Cells were seeded in a 6-well plate and the following day, plasmids of interest were transfected by using FuGene HD transfection reagent (Promega) according to the manufacturer’s instructions. After incubation, the culture medium was aspirated and then the cells lysed by the addition of 1x Laemmli buffer (2% SDS, 10% glycerol, 0.0025% bromophenol blue, 0.1 M DTT and 63 mM Tris, pH = 6.8). A Bio-Rad mini-protean 3 chamber apparatus was employed for SDS-PAGE and then protein samples were transferred to an ImmunBlot PVDF membrane (Millipore) by electroblotting. After primary and secondary antibody incubation, the Clarity Western ECL substrate (Bio-Rad) was added to the PVDF membrane and the result was visualized by using the ChemiDoc Imaging System (Bio-Rad). Images were viewed and evaluated using ImageLab software (Bio-Rad). The primary antibody and secondary antibodies used are listed in S4 Table.

### NanoBiT assay

The NanoLuc Binary Technology (NanoBiT) assay was performed by using the NanoBiT PPI MCS Starter System (Promega), which detects protein-protein interactions in living cells based on structural complementation between Large BiT (LgBiT; 18 kDa) and Small BiT (SmBiT; 11 amino acid peptide) subunits. *clock1a*, *bmal1a* and *6–4phr* cDNA sequences with their sub-deletion fragments, and *tefb* were fused with LgBiT or SmBiT at the N- or the C-terminus. To generate the various NanoBiT fusion plasmids, the coding sequences of all the proteins of interest were PCR amplified and then cloned into CMV-based vectors (pCS2-MTK or pcDNA3.1). All pairwise combinations were transiently transfected into zebrafish PAC-2 cells using FuGene HD transfection reagent (Promega) and the cells were incubated in darkness for 2 days. After addition of Nano-Glo Live Cell Reagent, a non-lytic detection reagent including the cell-permeable furimazine substrate, the bioluminescence signals were detected by a Topcount NXT automatic scintillation counter (Perkin Elmer).

### Yeast two-hybrid assay

The yeast two-hybrid assay was performed according to the user manual of the Matchmaker Gold Yeast Two-Hybrid System (Clontech Laboratories, Inc.). All the *clock1a* and *bmal1a* derived fragments, *6–4phr* and *tefb* were amplified by PCR and ligated with either the pGBKT7 or pGADT7 vectors. For all the constructs, the DNA-binding domain of GAL4 (BD) or activation domain of GAL4 (AD) was fused at the N- or C-terminus of targeted genes or their mutants. The BD and AD-based vectors were subsequently transformed into the yeast strains AH109 and Y187 which were mated for protein-protein interaction detection.

### Statistical analysis

All data were calculated and represented as mean ± SEM. Additionally, they were plotted by using GraphPad Prism 9 (GraphPad Software Inc.) and analyzed by SPSS Statistics 19.0 (IBM). To evaluate statistically significant differences, either a Student’s t-test or an analysis of variance (ANOVA) was employed, and then Sidak’s multiple comparison post-test was applied. Statistics were defined to be significant when the p value was less than 0.05 and the statistical differences of p < 0.05, p < 0.01, p < 0.001 are indicated by *, ** and ***, respectively.

## Supporting information

S1 Fig Clock genes expression in medaka WT and photolyase mutant cell lines. Medaka WT and photolyase mutant cell lines were maintained under light-dark cycle condition for 4 days to entrain standard circadian clock and thereafter sampled at regular timepoints through a complete light-dark cycle. The mRNA expression levels of light-driven gene *per2a* as well as clock-regulated genes *per1b* and *per3* were analyzed by qRT-PCR. *Zeitgeber* (zt) sampling timepoints are plotted on the x-axes, while relative mRNA expression calculated as mean ± SEM (n = 3) is plotted on the y-axes. All these assays were repeated individually at least 3 times. White and black bars below each panel denote the light and dark periods, respectively.
(TIF)

S2 Fig NanoBiT-tagged Clock1a, Bmal1a and Tefb protein expression and protein-protein interaction assays. **(a-c)** Western blotting analysis of NanoBiT-tagged expression constructs, including *clock1a*, *bmal1a*, *tefb* and *6–4phr*. The NanoBiT constructs were transfected into zebrafish PAC-2 cells and after 2 days incubation, the cells were harvested for subsequent SDS-PAGE. The antibodies used are listed on S4 Table. **(d, g)** NanoBiT assay in zebrafish PAC-2 cells to test the heterodimerization of N terminally, SmBiT-tagged Clock1a and C terminally, LgBiT-tagged Bmal1a and homodimerization of C terminally, LgBiT and SmBiT tagged Tefb. The PRKACA:PRKAR2A pair was used as a constitutive positive control, while the Halo Tag-SmBiT was used as a negative control. Bioluminescence (cps) values are plotted on the y-axis with the mean ± SEM. **(e-f, h)** Yeast 2 hybrid assay to test the expression in yeast cells of zebrafish Clock1a, Bmal1a, Tefb and 6–4phr. All combinations were transformed into the yeast strains, respectively and the transformants were patched onto agar plates including synthetic medium without leucine and tryptophan (LW). A titrated dilution was performed to denote the expression of each combination. All assays were performed at least 3 times.
(TIF)

S3 Fig 6–4phr, Clock1a and Bmal1a sub-deletions protein expression and protein-protein interaction assays. **(a-g)** Western blotting of LgBiT- or SmBiT-tagged 6–4phr, Clock1a and Bmal1a deletions to test the expression in zebrafish PAC-2 cells. All of these deletion constructs were transfected into PAC-2 cells and after 2 days, the cells were harvested for western blotting analysis. The antibodies used are listed on S4 Table. **(h-i)** Yeast 2 hybrid assay to test the expression in yeast cells of the zebrafish Clock1a deletions, Bmal1a deletions and full length of 6–4phr. All possible combinations were transformed into the yeast strains, respectively and the transformants were patched onto agar plates including synthetic medium without leucine and tryptophan (LW). A titrated dilution was performed to denote the expression of each combination. All assays were performed at least 3 times, independently.
(TIF)

S1 Table Primer list and efficiency for qRT-PCR in medaka WT and photolyase mutant cells and fin clips. (XLSX)

S2 Table Primer list for construction of NanoBiT assay plasmids. (XLSX)

S3 Table Primer list for construction of yeast two hybrid assay plasmids. (XLSX)

S4 Table Antibody list for western blotting. (XLSX)

S1 Data **Tab A: Fig 1a.** Clock gene *per1b* expression in medaka 6–4 wild type (WT) and mutant (KO) cell lines. **Fig 1b.** Clock gene *per2a* expression in medaka 6–4 wild type (WT) and mutant (KO) cell lines. **Fig 1c.** Clock gene *per3* expression in medaka 6–4 wild type (WT) and mutant (KO) cell lines. **Fig 1d.** Clock gene *per1b* expression in medaka explanted wild type (iCab) and 6–4phr mutant (6–4 KO) fish fin clips. **Fig 1e.** Clock gene *per2a* expression in medaka explanted wild type (iCab) and 6–4phr mutant (6–4 KO) fish fin clips. **Fig 1f.** Clock gene *per3* expression in medaka explanted wild type (iCab) and 6–4phr mutant (6–4 KO) fish fin clips. **Fig 1g.** *In vivo* luciferase assay of medaka 6–4 WT and KO cell lines transfected with a luciferase reporter carrying the zebrafish clock-regulated *per1b* promoter. **Fig 1h.** *In vitro* luciferase assay of medaka 6–4 WT and KO cells transfected with a luciferase reporter construct containing 15 tandemly repeated copies of the D-box sequence derived from the zebrafish *cry1aa* gene promoter (*D-box*_*cry1aa*_*Luc*) and exposed to visible light or maintained in constant darkness. **Tab B: Fig 2a.** *In vitro* luciferase assay of zebrafish PAC-2 cells co-transfected with an E-box luciferase reporter construct containing 4 tandemly multimerized copies of the E-box sequence derived from the zebrafish *per1b* gene promoter (*4xE-box (-7)*) and expression vectors for zebrafish Clock1a and Bmal1a, 6–4phr, CPDphr and Cry1aa. **Fig 2b.** *In vitro* luciferase assay of mammalian 3T3 cells co-transfected with an E-box luciferase reporter construct containing 4 tandemly multimerized copies of the E-box sequence derived from the zebrafish *per1b* gene promoter (*4xE-box (-7)*) and expression vectors for zebrafish Clock1a and Bmal1a, 6–4phr, CPDphr and Cry1aa. **Fig 2c.** *In vitro* luciferase assay of zebrafish PAC-2 cells co-transfected with a D-box reporter construct containing 15 tandemly multimerized copies of the D-box sequence derived from the zebrafish *cry1aa* gene promoter (*D-box*_*cry1aa*_*Luc*) together with expression vectors for the six PAR factors, specifically Tefa, Tefb, Dbpa, Dbpb, Hlfa and Hlfb as well as zebrafish 6–4phr. **Fig 2d.** *In vitro* luciferase assay of zebrafish PAC-2 cells co-transfected with a D-box reporter construct containing 15 tandemly multimerized copies of the D-box sequence derived from the zebrafish *cry1aa* gene promoter (*D-box*_*cry1aa*_*Luc*) together with expression vectors for the six E4bp4/nfil3 factors as well as zebrafish 6–4phr. **Fig 2e.** *In vitro* luciferase assay of zebrafish PAC-2 cells co-transfected with the *D-box*_*cry1aa*_*Luc* reporter construct and expression vector for zebrafish Tefb together with the titration of expression vector for 6–4phr. **Fig 2f.** *In vitro* luciferase assay of mammalian 3T3 cells co-transfected with the *D-box*_*cry1aa*_*Luc* reporter construct and expression vector for zebrafish Tefb together with the titration of expression vector for 6–4phr. **Fig 2g.** *In vitro* luciferase assay of medaka 6–4 WT and KO cell lines co-transfected with the *D-box*_*cry1aa*_*Luc* reporter construct and the expression vector for zebrafish Tefb. **Fig 2h.** *In vitro* luciferase assay of zebrafish PAC-2 cells co-transfected with the *D-box*_*cry1aa*_*Luc* reporter construct and expression vector for zebrafish Tefb together with the titration of expression vector for Cry3. **Fig 2i.** *In vitro* luciferase assay of zebrafish PAC-2 cells co-transfected with the *D-box*_*cry1aa*_*Luc* reporter construct and expression vector for zebrafish Tefb together with the titration of expression vector for Cry1aa. **Fig 2j.** *In vitro* luciferase assay of zebrafish PAC-2 cells co-transfected with the *D-box*_*cry1aa*_*Luc* reporter construct and expression vector for zebrafish Tefb together with the titration of expression vector for CPDphr. **Tab C: Fig 3a.** NanoLuc Binary Technology (NanoBiT) assay to test the physical interaction between zebrafish Clock1a and 6–4phr. **Fig 3b.** NanoLuc Binary Technology (NanoBiT) assay to test the physical interaction between zebrafish Bmal1a and 6–4phr. **Fig 3e.** NanoLuc Binary Technology (NanoBiT) assay to test the physical interaction between zebrafish Tefb and 6–4phr. **Tab D: Fig 4b.** NanoBiT assays testing which domains of 6–4phr mediate the interactions with the Clock1a proteins. **Fig 4c.** NanoBiT assays testing which domains of 6–4phr mediate the interactions with the Bmal1a proteins. **Fig 4d.** NanoBiT assays testing which domains of 6–4phr mediate the interactions with the Tefb proteins. **Tab E: Fig 5a.** NanoBiT assay to test the effect of 6–4phr on Clock1a-Bmal1a heterodimerization. **Fig 5d.** NanoBiT assay to test the domains of Clock1a responsible for the interaction with 6–4phr. **Fig 5e.** NanoBiT assay to test the domains of Bmal1a responsible for the interaction with 6–4phr. Tab F: - S1a Fig. Clock gene *per1b* expression in medaka CPD wild type (WT) and mutant (KO) cell lines. **S1b Fig.** Clock gene *per2a* expression in medaka CPD wild type (WT) and mutant (KO) cell lines. **S1c Fig.** Clock gene *per3* expression in medaka CPD wild type (WT) and mutant (KO) cell lines. **S1d Fig.** Clock gene *per1b* expression in medaka DASH wild type (WT) and mutant (KO) cell lines. **S1e Fig.** Clock gene *per2a* expression in medaka DASH wild type (WT) and mutant (KO) cell lines. **S1f Fig.** Clock gene *per3* expression in medaka DASH wild type (WT) and mutant (KO) cell lines. **Tab G: S2a Fig.** Western blotting analysis of NanoBiT-tagged expression constructs, including *clock1a*, *bmal1a* and *6–4phr.* **S2b Fig.** Western blotting analysis of NanoBiT-tagged expression constructs, including *clock1a*, *bmal1a and tefb.* **S2c Fig.** Western blotting analysis of NanoBiT-tagged *bmal1a* expression construct. **S2d Fig.** NanoBiT assay in zebrafish PAC-2 cells to test the heterodimerization of N terminally, SmBiT-tagged Clock1a and C terminally, LgBiT-tagged Bmal1a. **S2g Fig.** NanoBiT assay in zebrafish PAC-2 cells to test the homodimerization of C terminally, LgBiT and SmBiT tagged Tefb. **Tab H: S3a Fig.** Western blotting of LgBiT- or SmBiT-tagged 6–4phr deletions to test the expression in zebrafish PAC-2 cells. **S3b Fig.** Western blotting of LgBiT- or SmBiT-tagged 6–4phr deletions to test the expression in zebrafish PAC-2 cells. **S3c Fig.** Western blotting of LgBiT- or SmBiT-tagged Clock1a deletions to test the expression in zebrafish PAC-2 cells. **S3d Fig.** Western blotting of LgBiT- or SmBiT-tagged Clock1a deletions to test the expression in zebrafish PAC-2 cells. **S3e Fig.** Western blotting of LgBiT- or SmBiT-tagged Bmal1a deletions to test the expression in zebrafish PAC-2 cells. **S3f Fig.** Western blotting of LgBiT- or SmBiT-tagged Bmal1a deletions to test the expression in zebrafish PAC-2 cells. **S3g Fig.** Western blotting of LgBiT- or SmBiT-tagged Bmal1a deletions to test the expression in zebrafish PAC-2 cells.
(XLSX)
