# Supplementary figures and images for: 6-4 photolyase differentially modulates transcription in the vertebrate circadian clock
Source: PLoS Genet. 2025 Dec 12;21(12):e1011971. doi: 10.1371/journal.pgen.1011971 (PMC12716789; doi:10.1371/journal.pgen.1011971)

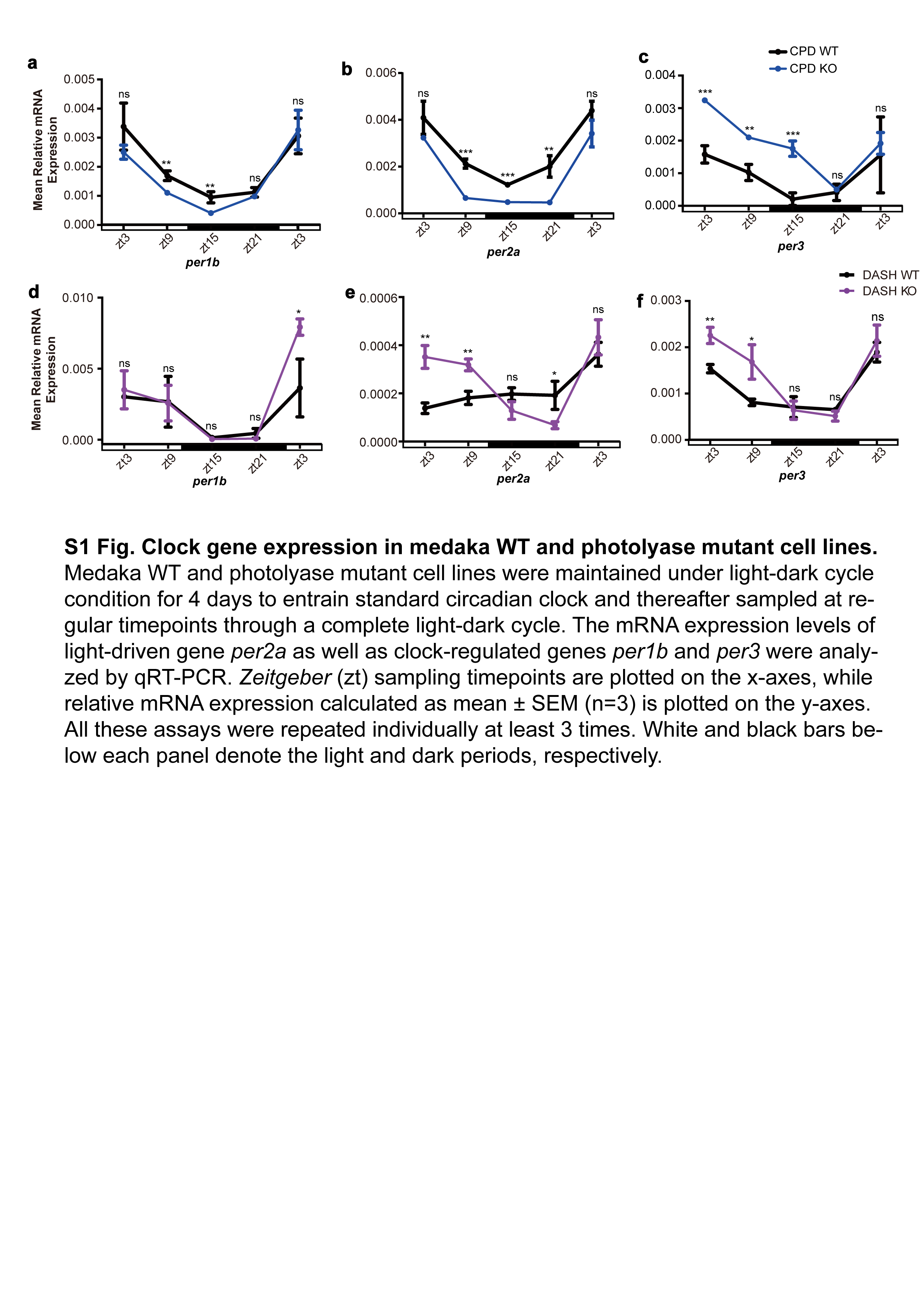

Supplement: S1 Fig — Medaka WT and photolyase mutant cell lines were maintained under light-dark cycle condition for 4 days to entrain standard circadian clock and thereafter sampled at regular timepoints through a complete light-dark cycle. The mRNA expression levels of light-driven gene per2a as well as clock-regulated genes per1b and per3 were analyzed by qRT-PCR. Zeitgeber (zt) sampling timepoints are plotted on the x-axes, while relative mRNA expression calculated as mean ± SEM (n = 3) is plotted on the y-axes. All these assays were repeated individually at least 3 times. White and black bars below each panel denote the light and dark periods, respectively. (TIF) [file pgen.1011971.s001.tif]

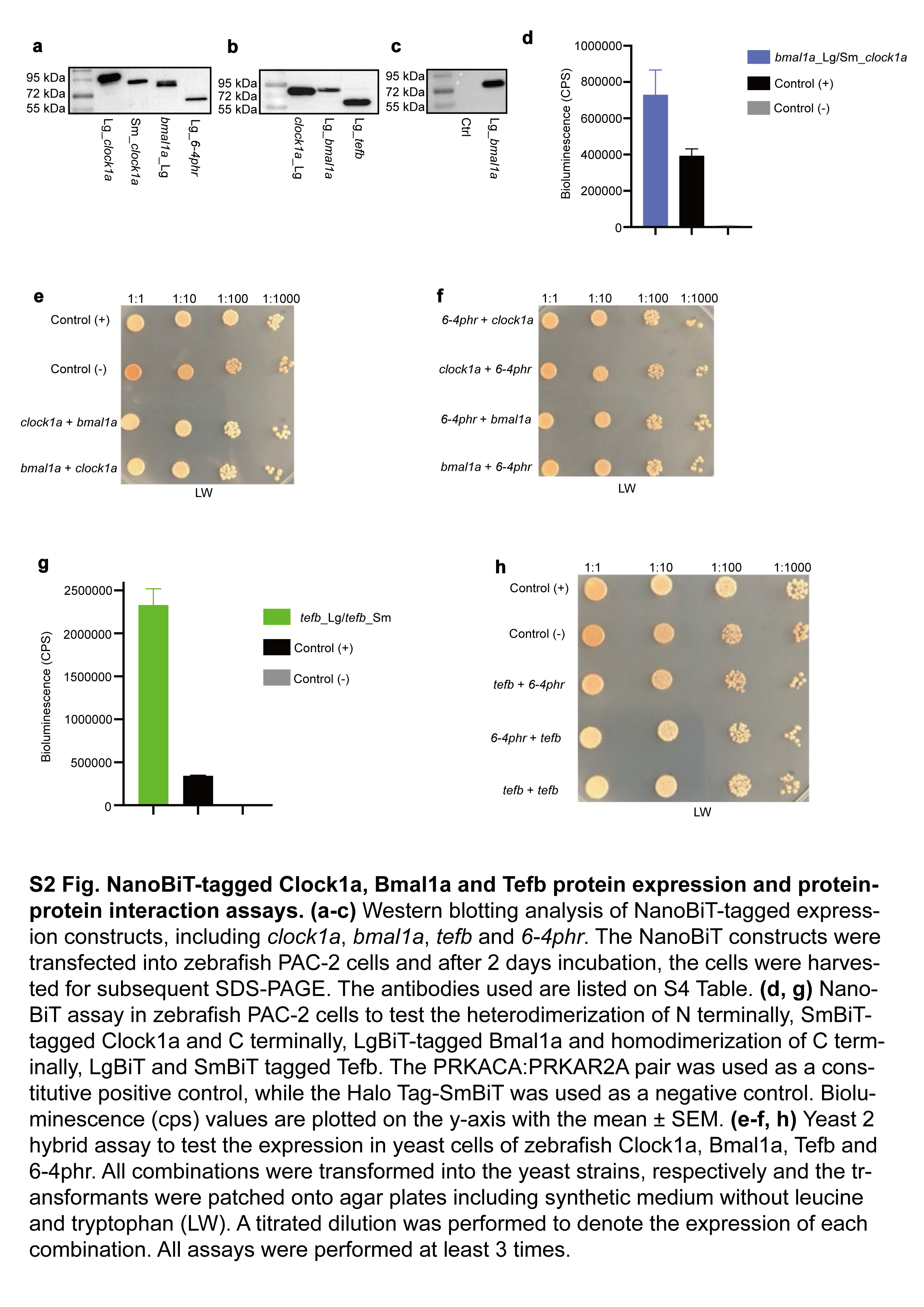

Supplement: S2 Fig — (a-c) Western blotting analysis of NanoBiT-tagged expression constructs, including clock1a, bmal1a, tefb and 6–4phr. The NanoBiT constructs were transfected into zebrafish PAC-2 cells and after 2 days incubation, the cells were harvested for subsequent SDS-PAGE. The antibodies used are listed on S4 Table. (d, g) NanoBiT assay in zebrafish PAC-2 cells to test the heterodimerization of N terminally, SmBiT-tagged Clock1a and C terminally, LgBiT-tagged Bmal1a and homodimerization of C terminally, LgBiT and SmBiT tagged Tefb. The PRKACA:PRKAR2A pair was used as a constitutive positive control, while the Halo Tag-SmBiT was used as a negative control. Bioluminescence (cps) values are plotted on the y-axis with the mean ± SEM. (e-f, h) Yeast 2 hybrid assay to test the expression in yeast cells of zebrafish Clock1a, Bmal1a, Tefb and 6–4phr. All combinations were transformed into the yeast strains, respectively and the transformants were patched onto agar plates including synthetic medium without leucine and tryptophan (LW). A titrated dilution was performed to denote the expression of each combination. All assays were performed at least 3 times. (TIF) [file pgen.1011971.s002.tif]

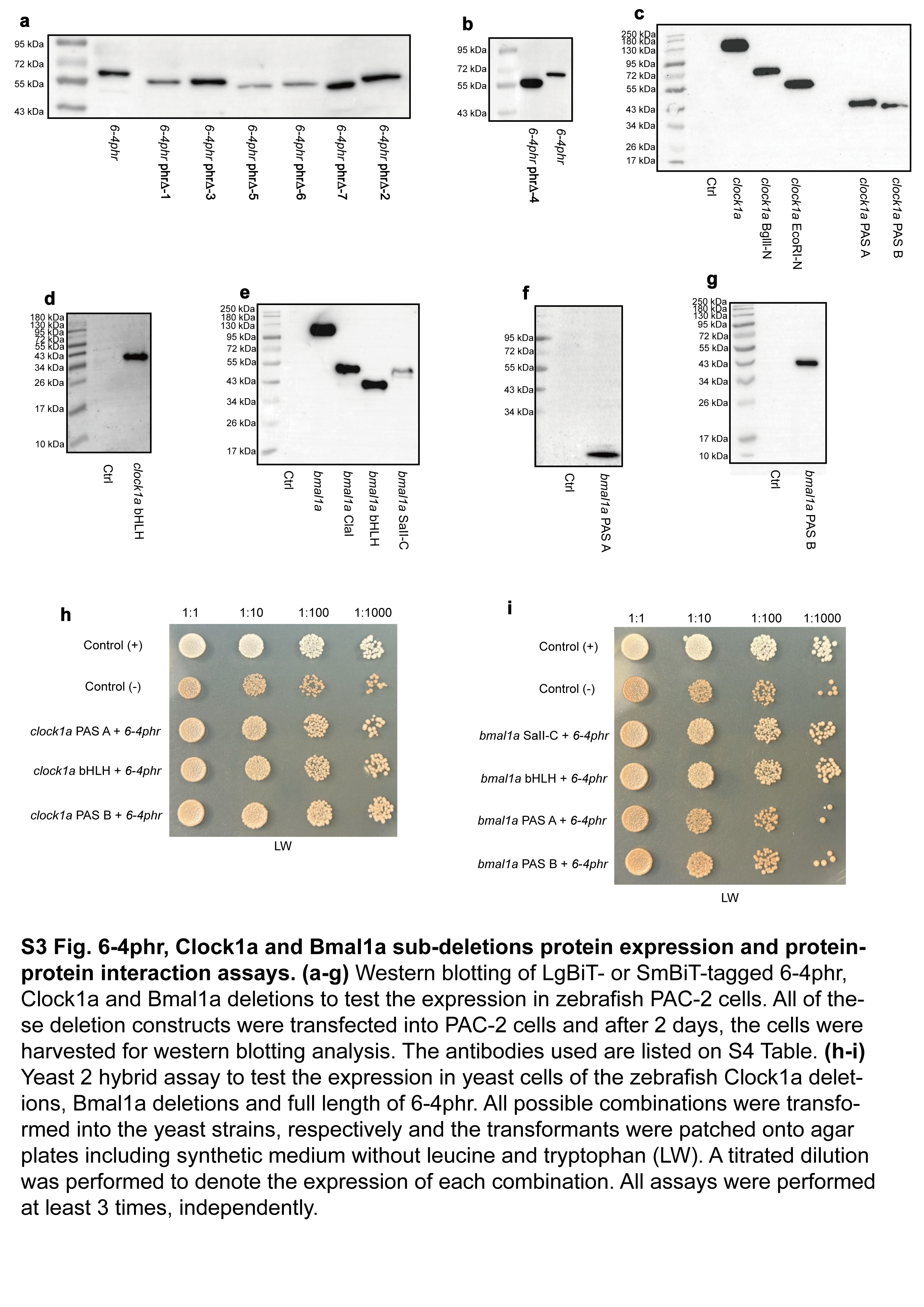

Supplement: S3 Fig — (a-g) Western blotting of LgBiT- or SmBiT-tagged 6–4phr, Clock1a and Bmal1a deletions to test the expression in zebrafish PAC-2 cells. All of these deletion constructs were transfected into PAC-2 cells and after 2 days, the cells were harvested for western blotting analysis. The antibodies used are listed on S4 Table. (h-i) Yeast 2 hybrid assay to test the expression in yeast cells of the zebrafish Clock1a deletions, Bmal1a deletions and full length of 6–4phr. All possible combinations were transformed into the yeast strains, respectively and the transformants were patched onto agar plates including synthetic medium without leucine and tryptophan (LW). A titrated dilution was performed to denote the expression of each combination. All assays were performed at least 3 times, independently. (TIF) [file pgen.1011971.s003.tif]
